# Measuring Blood Pressure in Mice using Volume Pressure Recording, a Tail-cuff Method

**DOI:** 10.3791/1291

**Published:** 2009-05-15

**Authors:** Alan Daugherty, Debra Rateri, Lu Hong, Anju Balakrishnan

**Affiliations:** Cardiovascular Research Center, University of Kentucky

## Abstract

The CODA 8-Channel High Throughput Non-Invasive Blood Pressure system measures the blood pressure in up to 8 mice or rats simultaneously. The CODA tail-cuff system uses Volume Pressure Recording (VPR) to measure the blood pressure by determining the tail blood volume. A specially designed differential pressure transducer and an occlusion tail-cuff measure the total blood volume in the tail without the need to obtain the individual pulse signal. Special attention is afforded to the length of the occlusion cuff in order to derive the most accurate blood pressure readings. VPR can easily obtain readings on dark-skinned rodents, such as C57BL6 mice and is MRI compatible. The CODA system provides you with measurements of six (6) different blood pressure parameters; systolic and diastolic blood pressure, heart rate, mean blood pressure, tail blood flow, and tail blood volume. Measurements can be made on either awake or anesthetized mice or rats. The CODA system includes a controller, laptop computer, software, cuffs, animal holders, infrared warming pads, and an infrared thermometer. There are seven different holder sizes for mice as small as 8 grams to rats as large as 900 grams.

**Figure:**
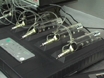


## Protocol

Equipment Set-up
  Select an area in the lab where the room temperature is at or above 20°C.
  Avoid locations near air conditioning vents and/or loud noise.
  Avoid exposing the animal to odors that may cause irritation and/or stress, such as perfume, cologne, chemicals, and cleaners.
  Turn on the CODA Controller.
  Turn on the Warming Platform and set to Level 3. If room temperature is above 25°C, start on Levels 1 or 2.
  Controller Diagnostics Test
    Open the CODA Software.
    Select the CODA Device by clicking on it.
    Click on “Test Selected Device”.
    Select the cuffs and channels to test and click “Test”.
    After the test is complete, close the Device Test window.
Software Set-up
  Manage Personnel and Animals
    Manage Personnel
      Select Tools > Manage Personnel.
      Click on “Researcher 1” to change the name.
      Click on subsequent rows to add additional researchers.
      Click on “Technicians Tab” to modify or add technicians.
      Click on “Specimens Tab” to name the animal for easy identification and to select the animal type; rat or mouse.
      Click on “Animals Tab” to alter the animal type and sensitivity.
  New Experiment
    Select File > New > Experiment
    Begin a New Experiment
      Enter the Experiment Name.
      Select the Key Researcher.
      Select a Begin Date and click “Next”.
    Basic Session Info
      Enter the Session Name
      Set “Acclimation Cycles” to 5. If you prefer not to include acclimation cycles, set to zero. To assist with acclimating the animal to the holder and cuffs, add additional cycles to your experiment and run the experiment for 5 consecutive days.
      Set “Number of Sets” to 1.
      Set “Time Between Sets” to 30 seconds for multiple sets
      Set “Cycles per Set” to 20 cycles.
      Set “Time Between Cycles” to 5 seconds and click “Next”.
  Specimen Selection
     
      Select the specimen from the specimen pool and assign it to the first available channel and click “Next”.
    Session Parameters
      Set “Maximum Occlusion Pressure” to 250mmHg.
      Set “Deflation Time” to 20 seconds for mice, 15 seconds for rats.
      Set “Minimum Volume” to 15ul.
      Set “Display Style” to One Channel per Graph.
      Add additional Researchers and Technicians and click “Next”.
    Session Script
      Review the Session Script and click “Next”.
      Do not select Finish until you complete the Animal Preparation section.
Animal Preparation
  Animal Set-up
    Animal Holder
      To train and improve animal acclimation, add additional cycles to your experiment and run the experiment for five consecutive days. You also can assist with acclimation by placing the animal in the holder for 15 minutes for 3 consecutive days prior to the actual study.
      Place each animal in a holder by picking up the animal by the tail and gently placing the animal into the rear of the holder which faces the open end of the nose cone. Note: the size of animal in relation to the size of the selected holder. The animal should be able to freely enter the holder.
      Carefully secure the rear hatch to the holder by turning the red screw cap on the rear hatch. Care should be taken to avoid pinching the tail or any other body parts while securing the rear hatch.
      Slide the nose cone toward the rear hatch, limiting the movement of the animal. The nose cone should be in a position to limit the animal from turning around while inside the holder.
      Place the holder onto the warming platform in the designated position. If testing the same group of animals over a period of time, be sure to rotate the holder position on the warming platform to improve consistency and accuracy of your BP measurements. Allow the animal at least 5-minutes to acclimate to the holder.
      Do not touch or handle the animal while inside the holder.  The increased contact could irritate the animal.
      Never leave the animal in the holders unattended.
    Cuff Placement
      Thread the tail through the “Occlusion Cuff.” Place this cuff as close to the base of the tail as possible without force.
      Secure the “Occlusion Cuff Tubing” in the notch on the top rear of the holder.
      Thread the tail through the “VPR Cuff”, placing it within 2mm of the “Occlusion Cuff”.
      Secure the “VPR Cuff Tubing” in the notch on the top rear of the holder.
      Attach the cuffs to the CODA Controller.
    Animal Temperature
      Allow the animal at least 5-minutes to thermoregulate.
      Record the temperature of the animal frequently with an infrared thermometer. The animal temperature should be 32 to 35°C.
Experiment
  Begin Experiment
    Click “Finish” and the experiment will begin immediately.
  End Experiment
    Animal
      Immediately remove the animal from the cuffs and holder.
      Return the animal to their cage after the “Simple Session Summary Report” is displayed.
    Data Collection
      After completion of the last cycle the accepted cycles will be automatically displayed in spreadsheet format within the CODA application, click on the “Export to Excel” button.
      Select the folder to place the file in, name the file and click “Save”.
      Open the Excel application and click on “Open”.
      View all file types to display the “.csv file saved”, click on the file.
    Data Processing
      There are several ways to process the data within the Excel file. A common practice is to obtain the average and standard deviation.
      Delete measurements if the standard deviation is greater than 30.
      Additionally, rejected cycles may be viewed and the entire database can be easily exported to Excel. Consult the User’s Manual for additional information.

